# The Epithelial Cell-Associated Gene PMAIP1 Serves as a Prognostic Biomarker for Lung Adenocarcinoma and Can Regulate the Stemness of Lung Cancer

**DOI:** 10.1155/sci/2896484

**Published:** 2025-11-21

**Authors:** Haoran Wang, Hui Zhang, Peipei Kang, Qin Ge, Xiaohong Chen, Gujun Cong

**Affiliations:** ^1^Department of Anesthesiology, Tumor Hospital Affiliated to Nantong University, Nantong, Jiangsu 226300, China; ^2^Department of Laboratory Medicine, Tumor Hospital Affiliated to Nantong University, Nantong, Jiangsu 226300, China; ^3^Department of Anesthesiology, First Affiliated Hospital of Soochow University, Suzhou, Jiangsu 226300, China; ^4^Department of Radiation Oncology, Tumor Hospital Affiliated to Nantong University, Nantong, Jiangsu 226300, China; ^5^Department of Laboratory Medicine, Nantong Fourth People's Hospital, Nantong, Jiangsu 226300, China

**Keywords:** epithelial cell-related gene, LUAD, machine learning, PMAIP1, single-cell analysis

## Abstract

Epithelial cells are integral to tumor composition and engage with various immune cell types within the tumor microenvironment, influencing tumor progression and metastasis. A thorough exploration of the roles and mechanisms of these epithelial cells could enhance early detection strategies and treatment modalities for lung adenocarcinoma (LUAD). This research employed single-cell analysis techniques, complemented by machine learning algorithms, to identify genes associated with epithelial cells and evaluate their prognostic significance and implications for immunotherapy in LUAD patients. By leveraging multiple datasets and applying diverse clustering methods within machine learning, we successfully crafted and validated a diagnostic model specifically for LUAD. Among the genes linked to epithelial cells, the XGBoost and random forest techniques identified PMAIP1 as the most crucial gene in terms of prognosis. Additionally, this study investigated the relationship between PMAIP1 and the infiltration of immune cells. The expression levels of PMAIP1 and its relevance in LUAD were subsequently confirmed through immunohistochemical staining and in vitro cell experiments. This analysis revealed 17 key genes associated with epithelial cells by integrating single-cell analysis with clinical data from the TCGA-LUAD dataset, underscoring their significance in diagnosis, prognostic assessment, and possible treatment avenues for LUAD patients. Importantly, PMAIP1 is strongly linked to prognosis and responses to immunotherapy in LUAD, with experimental findings indicating its heightened expression in PRAD and its connection to adverse outcomes. Furthermore, reducing PMAIP1 expression has been shown to hinder the proliferation, metastasis, and stemness of LUAD cells. In summary, our findings indicate that PMAIP1 has potential as a prognostic biomarker and a target for immunotherapy in patients with LUAD.

## 1. Introduction

Lung cancer is considered the most prevalent form of tumor worldwide and is the leading cause of cancer-related deaths [1, 2]. In 2018, approximately 1.8 million people globally succumbed to this disease [3]. A significant portion of lung cancer cases is linked to nonsmall cell carcinoma, with lung adenocarcinoma (LUAD) identified as the most common subtype [4, 5]. The complex biological functions and molecular mechanisms involved in tumor progression present considerable hurdles for the development of effective therapies and accurate prognostic evaluations [6]. Surgical intervention is the primary method for treating early-stage LUAD [7]. Nevertheless, many patients are diagnosed at a stage when the disease has either progressed locally or metastasized, necessitating systemic treatments such as chemotherapy, targeted therapies, or immunotherapy [8, 9]. Consequently, despite advancements in cancer research in recent years, the overall 5-year survival rate remains alarmingly low [10]. Therefore, it is vital to discover new targeted therapeutic agents to improve prognosis and treatment effectiveness for LUAD patients.

Epithelial cells are essential in the development and progression of tumors, having a significant impact on the tumor microenvironment and facilitating cancer advancement [11–14]. It is estimated that about 90% of malignant tumors arise from epithelial tissues, underscoring the significance of these cells in cancer biology studies. The structural arrangement of epithelial tissue features tight junctions between cells, which are upheld through various cell–cell interactions that are critical for maintaining cell polarity and ensuring tissue integrity. These junctions can shift to a state that encourages metastasis through a process known as epithelial-mesenchymal transition (EMT), during which epithelial cells lose their adhesive properties and gain the ability to migrate [15]. Epithelial cells can influence adjacent tumor cells in various ways. Under specific conditions, normal epithelial cells may generate signals that induce both phenotypic and genotypic changes in cancer cells, a phenomenon known as cancer cell redirection. This process can result in a transition from a malignant phenotype to one that promotes nontumorigenic epithelial characteristics. Such interactions suggest that a healthy epithelial microenvironment could inhibit the growth of tumorigenic cells [16]. Additionally, cancer cells participate in paracrine signaling with adjacent normal epithelial cells, which can promote tumor development and aid in the maintenance of cancer cells. For example, studies have shown that the glycoprotein THBS1 is crucial in facilitating paracrine interactions between cancer cells and normal epithelial cells within intestinal tumors. This interaction triggers developmental transcriptional programs in normal epithelial cells, supporting their survival within the tumor microenvironment and contributing to tumor growth [17]. Epithelial cells play a crucial role in influencing tumor characteristics, especially concerning their invasiveness. This influence mainly occurs through the secretion of extracellular matrix proteins and various signaling molecules, which help facilitate interactions with stromal cells [18]. The function of epithelial cells extends beyond merely aiding tumor formation through direct contact; they are essential in reshaping the tumor microenvironment, a critical process for the progression from a benign to a malignant state. Changes in the composition and mechanical properties of the extracellular matrix can activate numerous signaling pathways within tumor cells, promoting their proliferation, enhancing invasiveness, and contributing to treatment resistance. Epithelial cells play a crucial role in tumor biology, influencing not only the structural integrity of tumors, but also participating in the signaling pathways that are vital for tumor progression and metastasis. Their ability to influence tumor dynamics through both supportive and antagonistic interactions underscores the complexity of cancer, a disease fundamentally characterized by disrupted cellular communication [19]. Understanding these intricate interactions is vital for developing effective cancer therapies and for elucidating the mechanisms that drive tumor growth and dissemination.

Recent developments in technologies like single-cell RNA sequencing (scRNA-seq) and machine learning have provided new perspectives for the identification of biomarkers [20–22]. By analyzing individual cells, scientists can reveal the various cell types present in tissues, aiding in the discovery of distinct subpopulations of epithelial cells and their associated genes. Concurrently, machine learning utilizes extensive datasets to uncover potential biomarkers and their connections to clinical features, such as prognosis and reactions to immunotherapy [23]. By combining single-cell analysis with machine learning methods, we can accurately pinpoint functional genes linked to epithelial cells concerning LUAD prognosis and the efficacy of immunotherapy. Ultimately, this strategy enhances early detection and treatment approaches for this specific type of cancer.

## 2. Materials and Methods

### 2.1. Datasets and Patient Samples

This study focused on analyzing three LUAD samples (GSM4094260, GSM4094259, and GSM4094262) obtained from the GSE137912 dataset at the single-cell level [24]. Additionally, RNA sequencing data were integrated with clinical information from the TCGA-LUAD dataset. To develop and evaluate the diagnostic model, several datasets, including TCGA-LUAD, GSE63459, GSE43458, GSE40791, GSE32863, and GSE10072, were utilized [25–29]. Furthermore, this investigation gathered samples from patients diagnosed with LUAD who received surgical intervention at Nantong Tumor Hospital from June 2012 to March 2018. Each patient's follow-up period after surgery spanned from 1 to 6 years, with follow-ups extending until August 2019. All participants provided informed consent in writing, and the study received approval from the Ethics Committee of Nantong Tumor Hospital. After filtering out samples lacking complete clinical information or those without follow-up data, 90 LUAD tissue samples and 90 normal lung tissue samples were ultimately included in the analysis.

### 2.2. scRNA-Seq

The Seurat package was employed to construct objects and discard low-quality cells, thereby ensuring that only high-quality data were included in our analysis. We conducted a comprehensive preprocessing of the data, concentrating on the ratios of gene counts, cell counts, and mitochondrial content. Our criteria for filtering involved eliminating genes present in fewer than three cells and excluding cells that contained fewer than 200 genes. To achieve data standardization across various samples, we normalized the UMI counts for each cell using a scaling factor of 10,000. After performing a log transformation on the data, we utilized the ScaleData function from Seurat (v3.0.2) to enhance the quality of the normalized dataset. For principal component analysis (PCA), we selected the top 10 variable genes to pinpoint the main factors influencing variability in the dataset. In preparation for t-SNE visualization and clustering, we retained the first 11 principal components, which offered significant insights into the underlying structure of the data. The cell clustering was accomplished through the FindClusters function within the Seurat package, applying a resolution parameter of 0.5 to guarantee distinctly defined clustering patterns among the cells [20, 30].

### 2.3. Non-Negative Matrix Factorization (NMF) Cluster Analysis

The algorithm known as NMF was employed to pinpoint biologically significant coefficients within the gene expression matrix. This method not only efficiently organized genes and samples, but also illuminated the inherent structural features present in the data. By concentrating on these features, the algorithm supported the grouping of samples, which contributed to a more profound comprehension of the fundamental biological patterns and relationships among the analyzed samples [31]. To explore the differences in gene expression across various clusters, especially between clusters A and B, a differential expression analysis was conducted utilizing the R package “Limma.” In this analysis, specific criteria were established, including a log-fold change (|logFC|) threshold exceeding 0.5 and an adjusted *p*-value below 0.05, to ensure that only notable expression differences were included. After this initial examination, the “NMF” R package was employed once more to cluster all samples based on the differentially expressed genes (DEGs) identified within the subclusters. This clustering aimed to reveal potential molecular subtypes that could shed light on the biological processes occurring within the dataset. For the clustering operation, the “brunet” algorithm was applied, executing 100 iterations for each predetermined cluster count, ranging from 2 to 10 clusters. The ideal number of clusters was determined by evaluating several metrics, including cophenetic correlation, dispersion, and silhouette width [32].

### 2.4. Constructing Diagnostic Model

Various diagnostic models related to LUAD were developed through the combination of several machine learning methodologies. The training phase made use of the TCGA-LUAD dataset, while the validation process involved the datasets GSE63459, GSE43458, GSE40791, GSE32863, and GSE10072. Each model combination was evaluated based on its area under the curve (AUC) value, and the model with the highest average AUC was chosen as the best one. The analysis of the receiver operating characteristic (ROC) curve was conducted using the pROC package [1.18.0], and the outcomes were presented through ggplot2 [3.3.6].

### 2.5. Cell Culture

The A549 (catalog number CL-0016) and NCI-H1299 (catalog number CL-0165) nonsmall cell lung cancer (NSCLC) cell lines were obtained from Procell Life Science and Technology Co., Ltd. They were maintained in F-12K medium (catalog number iCell-0007; iCell) and RPMI-1640 medium (catalog number R8758; MilliporeSigma), respectively. Each medium contained 10% fetal bovine serum (FBS) along with 1% penicillin-streptomycin (P/S). All cell lines were incubated at 37°C in an environment with 5% CO_2_ and high humidity [33].

### 2.6. Quantitative Reverse Transcription PCR (qRT-PCR)

RNA was extracted from cells using Trizol reagent, after which cDNA was synthesized via reverse transcription with the RevertAid FirstStrand cDNA Synthesis Kit. The qRT-PCR was conducted using the Applied Biosystems 7900HT Fast Real-Time PCR System, following the reaction setup detailed below [34]. The following are the sequences of the primer pairs for the target genes: PMAIP1 (forward: GATGAGGAGCCCAAGCCCAACC, reverse: CCCAAACGACTGCCCCCATACAA) and GAPDH (forward: CGGAGTCAACGGATTTGGTCGTAT, reverse: AGCCTTCTCCATGGTGGTGAAGAC).

### 2.7. Colony Formation Assay

The cells were transferred into 6-well plates and allowed to incubate for 2 weeks. After this incubation phase, methanol was employed to fix the cells, which were subsequently stained using a 0.1% crystal violet solution. An Olympus microscope facilitated the observation and counting of the cells.

### 2.8. Transwell Assay

Cell motility and invasion were evaluated utilizing Transwell petri dishes, both with and without the inclusion of Matrigel (Corning, Inc.). To summarize, colon cancer cells that underwent transfection (2 × 10^4^) were allocated into 100 µL of serum-free medium (Gibco; Thermo Fisher Scientific, Inc.) placed in the upper chamber. Concurrently, 500 µL of DMEM supplemented with 10% serum (Shanghai ExCell Biology, Inc.) was added to the lower chamber. The upper chamber was then incubated for 24 h at 37°C in a 5% CO_2_ atmosphere, after which the cells were fixed with 4% paraformaldehyde (Beyotime Institute of Biotechnology) for 10 min at ambient temperature. Following this, the cells underwent staining using a solution of 0.2%–0.5% crystal violet (Sigma-Aldrich; Merck KGaA) for 10 min at room temperature, and were subsequently examined under an inverted optical microscope (Shanghai Optical Instrument Factory) for statistical analysis. The procedure for the migration assay closely resembled that of the invasion assay, with the primary distinction being the absence of Matrigel.

### 2.9. Immunohistochemical Analysis of PMAIP1 Expression in LUAD Tissue

The tissues were fixed in a 4% paraformaldehyde solution for 15 min, followed by embedding in paraffin and sectioning to an average thickness of 4 μm. After dewaxing and dehydration, antigen retrieval was performed. The resulting sections were treated with a 3% hydrogen peroxide solution for 20 min and subsequently blocked with a 5% BSA solution at room temperature for 15 min. The PMAIP1 antibody (CSB-PA969433) was then diluted in an antibody diluent (Life-iLab, Shanghai, China) and applied to the sections. The sections were developed using a chromogenic agent for 3–15 min. Following this, they were sequentially washed, restained, dehydrated, cleared, and sealed. The processed sections were examined using the SP kit (Solarbio, Beijing, China). Finally, the sections were observed and photographed under an optical microscope [35].

### 2.10. Statistical Analysis

The levels of PMAIP1 expression in normal lung tissues and LUAD tissues were evaluated through the Wilcoxon rank-sum test. For analyzing prognosis, the log-rank test was employed. A *p*-value below 0.05 was deemed statistically significant.

## 3. Result

### 3.1. Identification of Epithelial Cell-Related Genes

**Figure 1:**
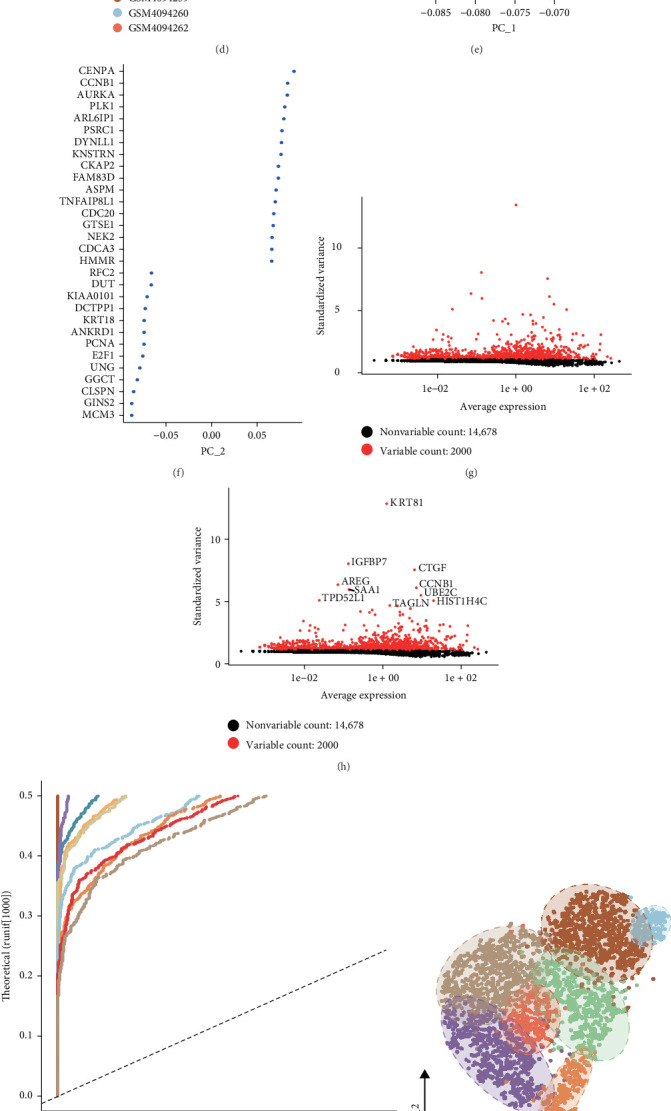
Identification of marker genes for epithelial cells. (A–C) Assessment of scRNA-seq quality among various cell subpopulations. (D–F) PCA analysis visualizations after the integration and removal of batch effects. (G, H) Identification of highly variable genes through batch removal conducted after counting. (I, J) Application of the t-SNE method for classifying LUAD samples. (K) Examination of the functions associated with different cell populations.

The research began with an examination of three LUAD samples obtained from the GSE137912 dataset, adhering to strict quality control criteria for cell selection. Each cell needed to have at least 1000 RNA molecules and not exceed 6000, with the stipulation that mitochondrial RNA made up no more than 15% of the overall RNA content (Figure 1A–C). Following this, we applied the rpca method to identify genes exhibiting significant variability in the filtered dataset, and then conducted assessments to remove batch effects based on the discovered feature sets (Figure 1D–F). The results from the ANOVA test identified 10 genes that showed notable differential expression across the cellular samples: KRT81, IGFBP7, CTGF, AREG, CCNB1, SAA1, UBE2C, TPD52L1, TAGLN, and HIST1H4C (Figure 1G–H). A single-cell analysis distinguished the three LUAD samples into seven distinct cell clusters: epithelial cells, B cells, multilymphoid progenitors, NTK cells, endothelial cells, neural progenitors, and microglia (Figure 1I–J). Importantly, functional analysis indicated that the epithelial cell cluster was associated with processes such as angiogenesis and the EMT (Figure 1K).

### 3.2. Functional Analysis of Epithelial Cell-Associated Genes

**Figure 2:**
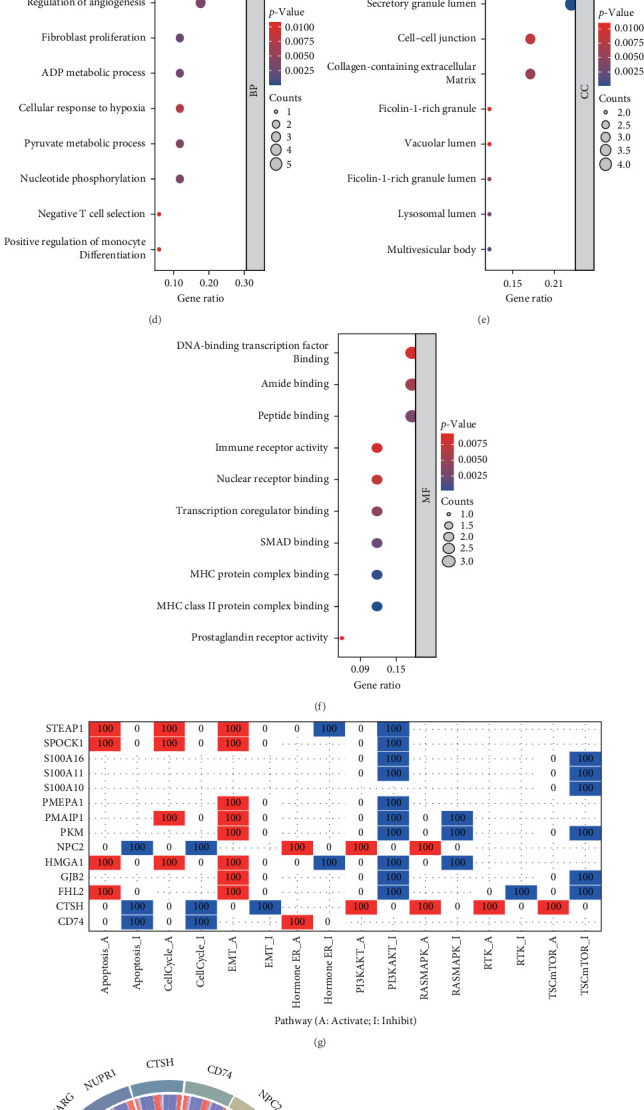
Analysis of epithelial cell functionality. (A) Comparative analysis. (B) Identification of prognostic genes related to epithelial cells. (C) The forest plot illustrates the prognostic variations in genes associated with epithelial cells. (D–F) Gene Ontology (GO) analysis. (G) Functional evaluation. (H) Correlation analysis of genes related to epithelial cells. (I) Heatmap displaying SNV percentages. (J) Variance in methylation. (K) Percentage of CNV.

Initially, a differential analysis was conducted utilizing cancerous samples alongside adjacent tissues sourced from the TCGA-LUAD dataset, while concurrently gathering prognostic genes. By identifying overlapping elements, we discovered 17 differentially prognostic genes associated with epithelial cells (Figure 2A, B). The forest plot demonstrates the prognostic differences among these 17 genes; notably, four of these genes function as protective prognostic factors for patients with LUAD, whereas the remaining 13 are classified as risk factors (Figure 2C). To further explore the functions of these 17 differentially prognostic genes tied to epithelial cells, we executed a Gene Ontology (GO) analysis. Our results reveal that these genes participate in the positive regulation of proteolysis, cellular reactions to peptides, the regulation of angiogenesis, and several lumen-related processes, such as vesicle lumen, cytoplasmic vesicle lumen, and secretory granule lumen, in addition to various interactions including DNA-binding transcription factor binding, amide binding, and peptide binding (Figure 2). Moreover, we accessed the Gene Set Cancer Analysis (GSCA) database—an essential tool for the functional analysis of gene sets—to scrutinize the roles of these genes in greater detail. We determined that the 17 differentially prognostic genes associated with epithelial cells mainly facilitate EMT while inhibiting the PI3K/AKT pathway (Figure 2G). Furthermore, we explored the connections among these 17 genes using a relevance chord diagram (Figure 2H). Finally, we examined the mutation and methylation statuses related to these genes.

### 3.3. Clustering Analysis

**Figure 3:**
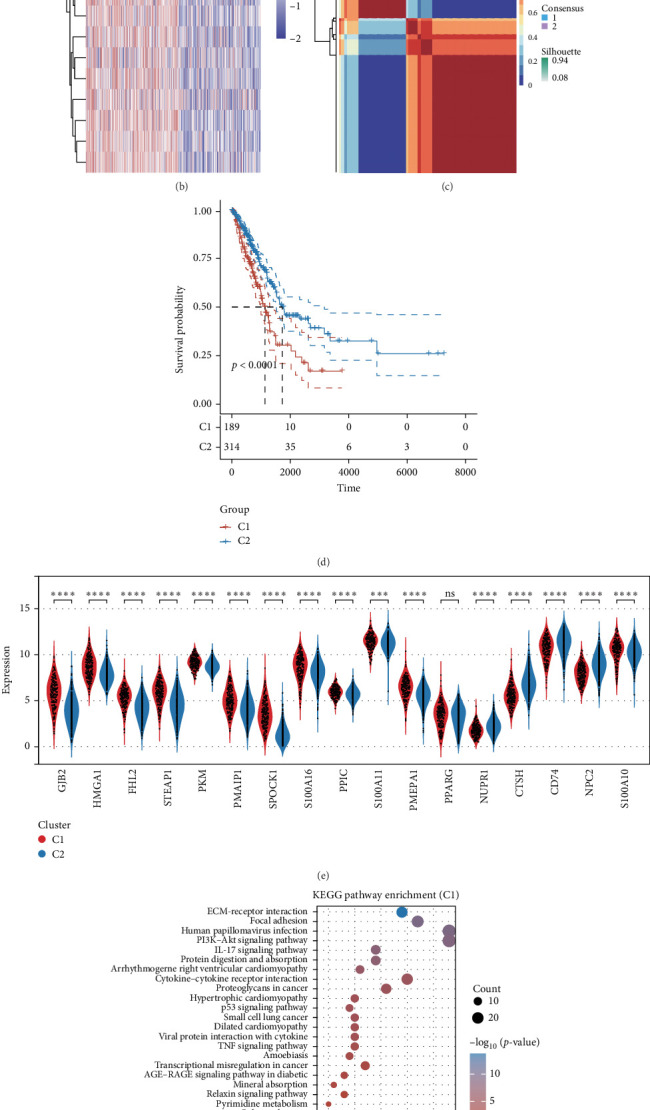
NMF cluster analysis. (A) Evaluation of the clusters' effectiveness and stability using various methods. (B, C) Consensus diagram depicting the outcomes of NMF clustering. (D) Survival rate differences among the distinct clusters. (E) Changes in gene expression linked to epithelial cells across the various clusters. (F, G) Analysis using KEGG. *⁣*^*∗∗∗*^*p* < 0.001; *⁣*^*∗∗∗∗*^*p* < 0.0001; ns *p＞*0.05.

To classify the TCGA-LUAD samples, we applied the clustering method known as NMF. We evaluated the coexpression profiles to determine the best approach for dividing the TCGA-LUAD sample subgroups. The optimal segmentation was indicated by the point on the curve where the coexpression index showed its steepest drop. Our findings illustrated that the division of TCGA-LUAD samples into two distinct groups was the most suitable method (Figure 3A). Following this, we represented the differences in gene expression across the various clusters using heatmaps (Figure 3B, C). After creating the two clusters, cluster 1's prognosis was significantly poorer compared to that of cluster 2 (Figure 3D). Importantly, the violin plots offered a more precise visualization of the expression differences for these genes among the clusters, with the exception of PPARG, which did not exhibit a notable expression difference between the two groups (Figure 3E). To investigate the possible mechanisms underlying the differences noted between cluster 1 and cluster 2, we performed KEGG enrichment analysis. The outcomes indicated that cluster 1 was linked to pathways including human papillomavirus infection, the PI3K−Akt signaling pathway, interactions between cytokines and their receptors, ECM−receptor interaction, and the p53 signaling pathway, whereas cluster 2 was associated with the PPAR signaling pathway, Ras signaling pathway, Wnt signaling pathway, glycerophospholipid metabolism, and glutathione metabolism (Figure 3F, G).

### 3.4. Analysis of the Correlation Between Epithelial Cell-Related Genes and Immunotherapy and Chemotherapy

**Figure 4:**
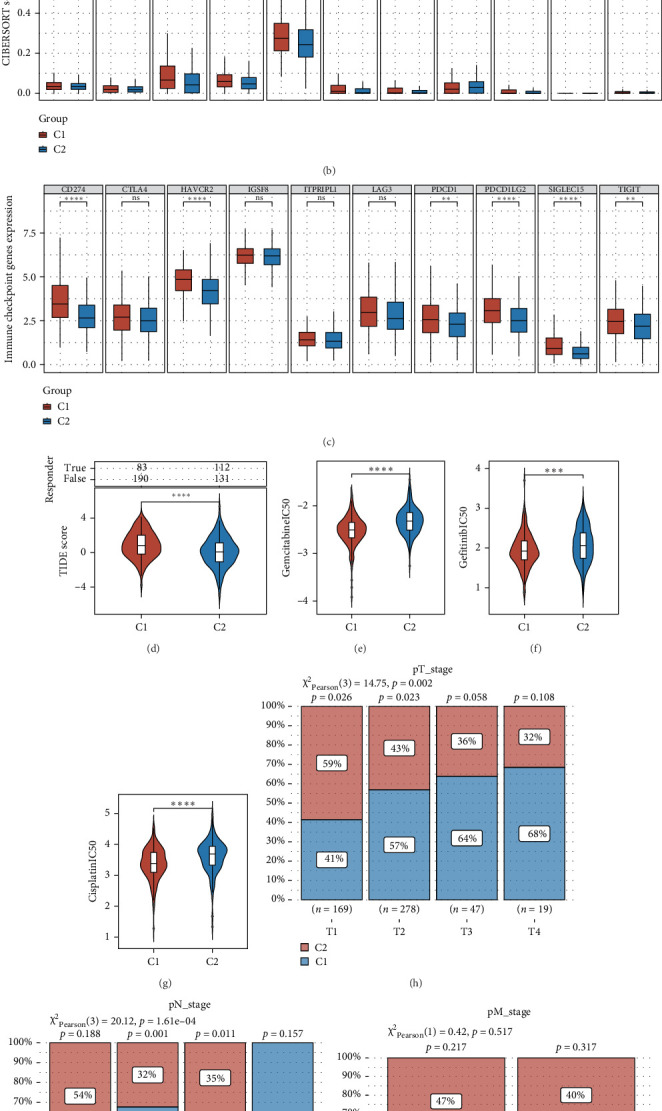
Genes related to epithelial cells are connected to the infiltration of immune cells. (A, B) This research explores the association between genes linked to epithelial cells and the invasion of immune cells. (C) The changes in the expression levels of immune checkpoint genes across diverse clusters are shown. (D) The evaluation of patient responses to immune checkpoint therapies across different clusters is performed using the TIDE algorithm. (E–G) The discrepancies in IC50 scores of various drugs among different clusters are investigated. (H–J) The differences in the distribution of various subgroups throughout different pathological stages of LUAD are examined. *⁣*^*∗*^*p* < 0.05; *⁣*^*∗∗*^*p* < 0.01; *⁣*^*∗∗∗*^*p* < 0.001; *⁣*^*∗∗∗∗*^*p* < 0.0001; ns *p＞*0.05.

To evaluate the connection between the epithelial cell-related genes we discovered and immunotherapy outcomes for LUAD patients, we analyzed the expression changes of 22 immune cell types across various clusters utilizing the CIBERSORT algorithm. Our results revealed a notable increase in the infiltration levels of plasma B cells, resting CD4+ memory T cells, and follicular helper T cells in cluster 2 compared to those in cluster 1. Conversely, cluster 1 demonstrated enhanced infiltration levels of activated CD4+ memory T cells, resting myeloid dendritic cells, and M0, M1, and M2 macrophages in relation to cluster 2 (Figure 4A, B). Subsequently, we investigated the expression variances of several immune checkpoint-related genes across the clusters. Our findings showed that CD274, HAVCR2, PDCD1, PDCD1LG2, SIGLEC15, and TIGIT exhibited higher expression levels in cluster 1 (Figure 4C). The tumor immune dysfunction and exclusion (TIDE) algorithm forecasts how specific samples or subtypes may react to immune checkpoint inhibitors. By applying this algorithm, we found that TIDE scores in patients from cluster 1 were significantly elevated compared to those in cluster 2, implying that the former group may face diminished efficacy in response to immune checkpoint blockade therapy (Figure 4D). Furthermore, we evaluated the IC50 scores for three medications—Gefitinib, Cisplatin, and Gemcitabine—across the various clusters. Our analysis indicated that IC50 scores for these drugs in cluster 2 were substantially higher than those in cluster 1 (Figure 4). In addition, we examined the distribution of patients at varying clinical stages across the clusters. The findings revealed significant disparities in T stage and N stage between cluster 1 and cluster 2 (Figure 4).

### 3.5. Constructing Diagnostic Model

**Figure 5:**
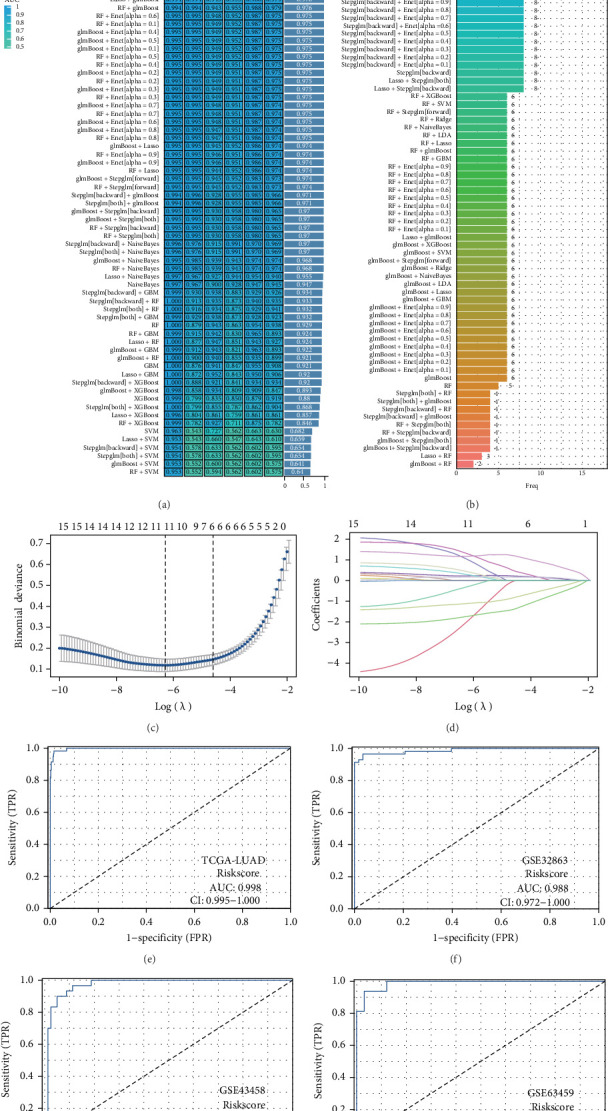
The diagnostic model constructed using the LASSO algorithm exhibits excellent predictive value. (A) An analysis of how stem cell-related genes can forecast diagnoses in patients with LUAD. (B) AUC metrics for diagnostic models formulated from different algorithmic combinations. (C–J) Build and assess a diagnostic model leveraging the LASSO algorithm.

To investigate the role of genes associated with epithelial cells in LUAD, we assessed their potential as predictive markers for diagnosing LUAD in patients using various machine learning methodologies. Our research focused on developing diagnostic models utilizing six datasets: the TCGA-LUAD dataset for training, along with GSE63459, GSE43458, GSE40791, GSE32863, and GSE10072 for validation purposes. Among the various algorithm combinations analyzed, the LASSO method emerged as the most effective for model development, achieving an AUC of 0.996 in the training dataset (TCGA-LUAD) and AUCs of 0.99, 0.968, 0.989, 0.992, and 0.985 across the validation datasets (GSE63459, GSE43458, GSE40791, GSE32863, and GSE10072), respectively. Overall, the diagnostic model produced an average AUC of 0.986 when evaluated across all five datasets, demonstrating strong predictive capability (Figure 5A). The LASSO-derived model incorporated 11 genes linked to epithelial cells: STEAP1, S100A11, S100A10, PPIC, PPARG, PMAIP1, PKM, NUPR1, HMGA1, FHL2, and CD74 (Figure 5B). Subsequently, we developed and validated a diagnostic model using these 11 genes through the LASSO algorithm, yielding encouraging results (Figure 5C–J).

### 3.6. PMAIP1 Was Identified by Us as a Key Prognostic Gene in Epithelial Cells

**Figure 6:**
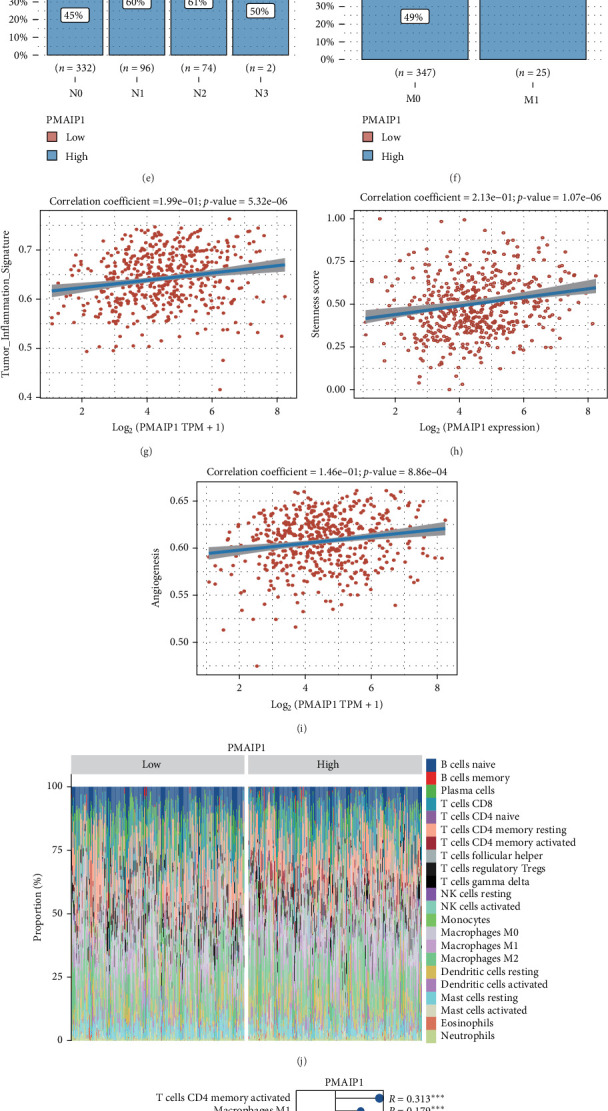
PMAIP1 is the most critical gene for LUAD prognosis. (A) The XGBoost algorithm identifies the 10 genes that show the most significant associations with overall survival (OS). (B) The random forest algorithm identifies 14 genes that are most strongly correlated with OS. (C) Gosemsim analysis reveals crucial genes tied to functions related to epithelial cells. (D–F) The TCGA-LUAD dataset showcases the differential expression of genes associated with epithelial cells across different staging levels. (G–I) A functional analysis of PMAIP1 is presented. (J, K) The relationship between PMAIP1 and immune infiltration in LUAD is examined. (L) An examination of the correlation between PMAIP1 and genes related to immune checkpoints is conducted. (M) The correlation between PMAIP1 expression and the effectiveness of immunotherapy in LUAD patients is analyzed. *⁣*^*∗*^*p* < 0.05; *⁣*^*∗∗*^*p* < 0.01; *⁣*^*∗∗∗*^*p* < 0.001; *⁣*^*∗∗∗∗*^*p* < 0.0001; ns *p＞*0.05.

We investigated the genes associated with epithelial cells in TCGA-LUAD samples by utilizing both the XGBoost and randomForest algorithms to analyze their relationship with overall survival (OS). In our findings from the XGBoost and randomForest analyses, PMAIP1 consistently obtained the top ranking (Figure 6A, B). Using Gosemsim analysis, we organized these stem cell-related genes according to their GO similarities, again resulting in PMAIP1 holding the first position (Figure 6C). We also assessed the variations in PMAIP1 expression across different staging categories within the TCGA-LUAD dataset, discovering that its expression correlated with the N and M stages in patients with LUAD (Figure 6). Following this, we applied the ssGSEA algorithm to compute the enrichment scores for each sample across multiple pathways in the TCGA-LUAD dataset, thus linking samples to specific pathways. By analyzing the correlation between PMAIP1 expression and pathway scores, we identified the relationship between PMAIP1 and various pathways. Our analysis revealed that PMAIP1 expression is associated with tumor inflammation, angiogenesis, and EMT (Figure 6). We additionally examined the relationship between PMAIP1 and immune infiltration in LUAD through the application of the CIBERSORT algorithm. Our findings indicated a positive association between PMAIP1 expression and the infiltration levels of activated CD4 memory T cells, M1 macrophages, gamma delta T cells, M2 macrophages, eosinophils, and resting dendritic cells. Conversely, we observed a negative correlation with the infiltration levels of activated NK cells, regulatory T cells (Tregs), resting mast cells, naive B cells, and plasma cells (Figure 6J, K). Furthermore, we categorized TCGA-LUAD samples according to PMAIP1 expression and assessed their relationship with genes related to immune checkpoints. The analysis revealed significant differences in the expression of all immune checkpoint-related genes between groups with high and low PMAIP1 expression (Figure 6L). Lastly, utilizing the TIDE algorithm, our results showed that patients exhibiting high PMAIP1 expression experienced less favorable outcomes when treated with immune checkpoint inhibitors (Figure 6M).

### 3.7. PMAIP1 is Highly Expressed in LUAD

**Figure 7:**
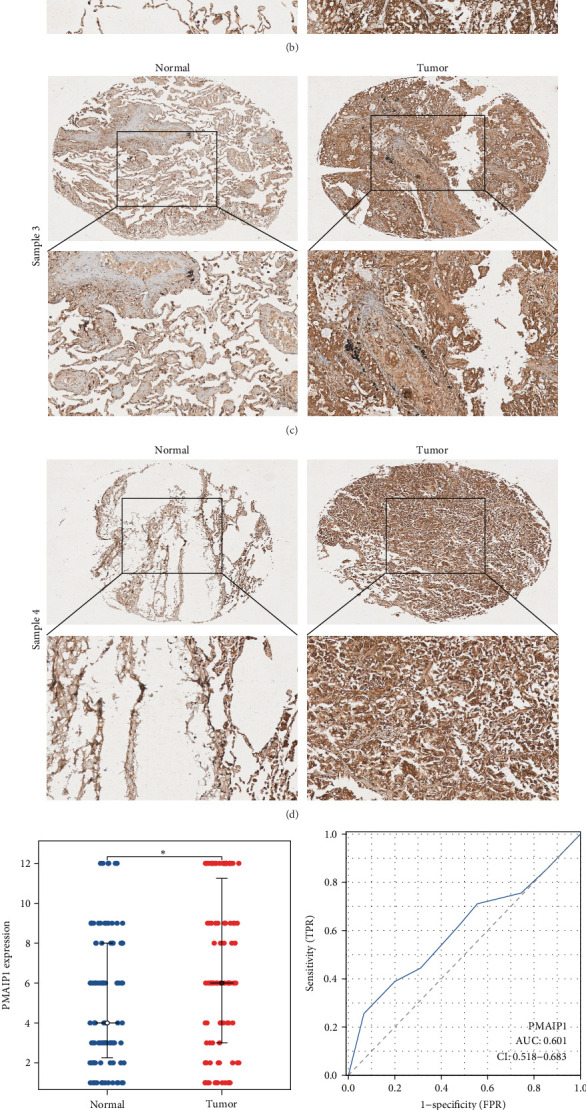
PMAIP1 can serve as a prognostic marker for LUAD patients. (A–E) The expression levels of PMAIP1 in LUAD exhibit differential patterns. (F) The ROC curve illustrates the potential of PMAIP1 as a diagnostic marker for LUAD. (G) The KM curve reflects the prognostic significance of PMAIP1. (H) The ROC curve highlights the role of PMAIP1 as a prognostic marker for LUAD. (I, J) Analysis of the correlation between PMAIP1 expression and various stages in patients with LUAD. *⁣*^*∗*^*p* < 0.05.

In order to further validate the expression levels and prognostic significance of PMAIP1 in LUAD, we collected 90 paired tissue samples from LUAD patients for subsequent examination. The evaluation of PMAIP1 expression in LUAD was performed through immunohistochemical staining (Figure 7), with scatter plots demonstrating the differences in PMAIP1 expression levels. Our results indicated that PMAIP1 levels in LUAD were significantly higher compared to normal lung tissues (Figure 7E). The ROC curve underscored the diagnostic capability of PMAIP1 for LUAD, revealing an AUC of 0.601 (Figure 7F). Moreover, we investigated the prognostic relevance of PMAIP1 in LUAD, finding that patients with elevated PMAIP1 expression encountered a worse prognosis, suggesting that PMAIP1 may act as a prognostic biomarker for those diagnosed with LUAD (Figure 7G, H). Finally, we examined the correlation between PMAIP1 expression and LUAD staging, identifying a connection between PMAIP1 levels and both T stage and N stage (Figure 7I, J).

### 3.8. PMAIP1 Can Affect the Proliferation and Metastasis of Lung Cancer Cells

**Figure 8:**
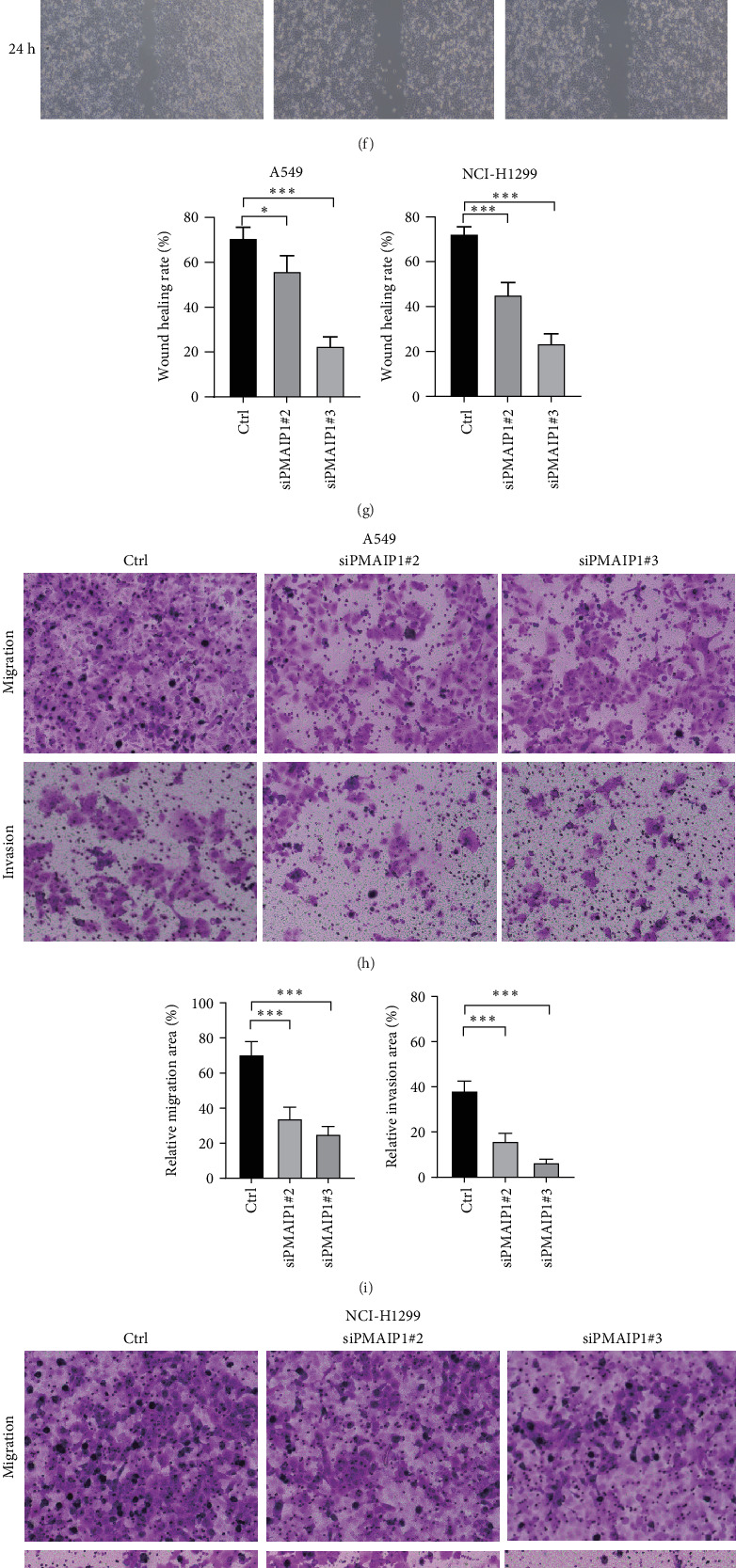
The inhibition of lung cancer cell proliferation and metastasis is achieved by knocking down PMAIP1. (A, B) qRT-PCR was employed to assess the efficiency of PMAIP1 siRNA knockdown. (C, D) A clone formation assay was conducted to evaluate the influence of PMAIP1 knockdown on cell proliferation. (E–G) The scratch healing assay was utilized to investigate the effect of PMAIP1 knockdown on cell migration. (H–K) The effects of PMAIP1 knockdown on cell migration were further analyzed using transwell assays. (L, M) The sphere formation assay was conducted to analyze the impact of PMAIP1 knockdown on the sphere-forming ability of A549 and NCI-H1299 cells. *⁣*^*∗*^*p* < 0.05; *⁣*^*∗∗∗*^*p* < 0.001; ns *p＞*0.05.

We evaluated the inhibitory effects of various PMAIP1 siRNA target sites using qRT-PCR technology. Our findings revealed that siPMAIP1#2 and siPMAIP1#3 exhibited the strongest inhibitory effects (Figure 8A, B). Consequently, these two target sites were selected for further cellular experiments. In the A549 and NCI-H1299 cell lines, we found that silencing PMAIP1 significantly inhibited cell proliferation (Figure 8C, D). Additionally, both scratch assay and transwell assay demonstrated that knockdown of PMAIP1 significantly reduced the migration and invasion capabilities of A549 and NCI-H1299 cells (Figure 8). Finally, we also analyzed the impact of PMAIP1 knockdown on the sphere-forming ability of A549 and NCI-H1299 cells through sphere formation assays. We found that knockdown of PMAIP1 significantly inhibited the sphere-forming ability of the tumor cells (Figure 8).

## 4. Discussion

According to information provided by the World Health Organization, lung cancer ranks among the most common and deadly types of malignant tumors globally, with NSCLC accounting for approximately 80%–85% of lung cancer cases [36]. LUAD, recognized as the most widespread subtype of NSCLC, is marked by the uncontrolled growth of lung tissue cells and is linked to a high rate of mortality. Without intervention, cancer cells are likely to continue their proliferation and spread to other tissues beyond the lungs. The lack of accurate methods for forecasting disease progression frequently results in many lung cancer patients not receiving timely and suitable treatment, leading to an overall 5-year survival rate of under 20% [37]. This research focuses on identifying genes associated with epithelial cells using single-cell analysis and machine learning techniques, evaluating their significant impact on the diagnosis, prognosis, and immunotherapy of LUAD.

The emergence of single-cell technology has revolutionized our understanding of LUAD by illuminating tumor heterogeneity with exceptional clarity. By leveraging transcriptomic data at the single-cell level, scRNA-seq reveals the complex dynamics within TME and the interactions among various cell types, which are essential for identifying biomarkers and shaping therapeutic approaches in LUAD. For example, studies demonstrate that scRNA-seq can elucidate the impact of intratumoral heterogeneity on treatment resistance, thereby facilitating the investigation of interactions between tumor and immune cells and identifying specific cell populations associated with poor prognosis [38, 39]. Techniques in machine learning have emerged as crucial additions to single-cell analysis, aiding in the identification of possible biomarkers by evaluating extensive datasets obtained from scRNA-seq and various other modalities. These algorithms excel at detecting patterns within intricate biological information, which allows for forecasts regarding disease advancement and responses to treatments. For example, models that employ decision tree methodologies have been used to classify different subtypes of lung cancer based on microRNA expression patterns, effectively differentiating between LUAD and lung squamous cell carcinoma [40]. By combining single-cell transcriptomic data with machine learning algorithms, researchers can create predictive models that reflect tumor diversity and the organization of the immune landscape. For instance, recent research utilizing large datasets has uncovered malignant epithelial cell populations associated with a worse prognosis, revealing activated pathways in advanced LUAD, including EMT [41]. The combination of single-cell technology with machine learning techniques creates an intriguing approach for deciphering the complexities associated with LUAD. By clarifying the spatial and molecular details of tumors, scientists can enhance the development of novel biomarkers that facilitate early diagnosis, increase prognostic precision, and tailor treatment strategies for each patient, thus promoting the use of personalized medicine in lung cancer treatment.

In our research, we performed an extensive evaluation of the importance of genes related to epithelial cells in the prognosis and immunotherapy for patients with LUAD. By developing a diagnostic model, we validated the relevance of the epithelial cell-related genes identified as markers for diagnosis. Ultimately, we found that PMAIP1 stands out as the most significant prognostic gene among those examined. Earlier studies, by integrating multiomics analyses and experimental validation, have indicated that PMAIP1 may play a role in regulating glucose metabolism, thus affecting the tumor microenvironment in breast cancer [42]. Furthermore, research has shown that PMAIP1 influences the development of follicular thyroid carcinoma through the Wnt3/FOSL1 signaling pathway [43]. Nonetheless, the involvement of PMAIP1 in LUAD, especially as a gene associated with epithelial cells, has not yet been documented. Using a LUAD tissue microarray and cell-based experiments, we established the important function of PMAIP1 in LUAD.

## 5. Conclusion

This study employs single-cell analysis in conjunction with various machine learning techniques to reveal a significant connection between genes associated with epithelial cells and factors such as prognosis, diagnosis, and immune cell infiltration in patients with LUAD. Importantly, PMAIP1 is identified as a critical marker for epithelial cells, strongly correlated with diagnostic and prognostic patterns, as well as immune infiltration in LUAD patients. In conclusion, this research offers vital insights that aid in the early detection of LUAD and enhance patient outcomes through the identification of novel biomarkers and potential therapeutic targets.

## Data Availability

The data that support the findings of this study are available from the corresponding author upon reasonable request.
